# Supplementary material for: Attitudes and Practices Related to COVID-19 Vaccination with the Second Booster Dose among Members of Athens Medical Association: Results from a Cross-Sectional Study
Source: Vaccines (Basel). 2023 Sep 12;11(9):1480. doi: 10.3390/vaccines11091480 (PMC10534426; doi:10.3390/vaccines11091480)
Supplement: Supplementary file 1 [file vaccines-11-01480-s001.zip › vaccines-2566609-supplementary.pdf]

Attitudes and Practices Related to COVID-19 vaccination with the second booster dose (fourth dose)

We invite you to voluntarily participate in this anonymous online survey conducted by Athens Medical Association regarding your attitudes and practices related to vaccination against COVID-19 with the fourth (booster) dose. By completing the anonymous questionnaire below, I declare that I consent to the data of my answers being used for the purpose of the study.

<u>Demographics</u>

1. Gender

Female
Male

2. Age (years)

3. Employment status

Private sector
Working in N.H.S.
Working with the Greek Army
Working in universities

<u>Attitudes and practices regarding vaccinations</u>

4. The vaccines are important for Public Health

Fully Agree
Agree
Disagree
Fully Disagree

5. In general, vaccines are safe.

Fully Agree
Agree
Disagree
Fully Disagree

6. In general, vaccines are effective.

Fully Agree
Agree
Disagree
Fully Disagree

7. I am concerned over vaccination side effects.

Fully Agree
Agree
Disagree
Fully Disagree

8. Information received from the Greek Public Health Authorities regarding COVID-19 vaccination with the fourth dose were reliable.

Fully Agree
Agree
Disagree
Fully Disagree

9. What was your source of information regarding the fourth dose of COVID-19 vaccine? (One answer)

Website of A.M.A.
Scientific Journals
(WHO, CDC, ECDC
Television/Radio
Social Media
Website of N.H.S.
Independent websites

10. Have you been vaccinated against COVID-19 with the fourth dose?

Yes
No

11. If no, explain why. One answer

Non-obligation of the fourth dose
History of 3 doses of the vaccine
Insufficient information for the fourth dose

12. Which of the following factors affected your opinion regarding the uptake of COVID-19 vaccine? (One answer)

Fear of side effects
I am not at risk of COVID-19 disease
The time of the development of the vaccines was short
Receiving homeopathy medication
Pending vaccination appointment
I oppose to vaccinations

13. Are you willing to do the fourth dose of COVID-19 vaccine?

Yes
No

14. Have you been vaccinated with the influenza vaccine for season 2022-2023?

Yes
No

15. Do you agree with the initial mandatory vaccination schedule against COVID-19 for medical personnel to protect public health?

Yes
No
